# Ocular Sarcoidosis Limited to Retinal Vascular Ischemia and Neovascularization

**DOI:** 10.7759/cureus.839

**Published:** 2016-10-21

**Authors:** Gawain Dyer, Austin Rohl, Saad Shaikh

**Affiliations:** 1 Department of Ophthalmology, Howard University College of Medicine; 2 Medicine, UCF College of Medicine; 3 Ophthalmology, UCF College of Medicine; 4 Ophthalmology, USF College of Medicine; 5 Ophthalmology, Howard University College of Medicine; 6 Ophthalmology, University of Texas Medical Branch at Galveston; 7 Ophthalmology, Orlando Veterans Affairs Medical Center; 8 Ophthalmology, Florida State University College of Medicine

**Keywords:** retinal neovascularization, ocular sarcoidosis, sarcoidosis, retina, ophthalmology, retinal ischemia, retinopathy

## Abstract

A 59-year-old Caucasian male experienced progressive vision loss secondary to retinal vascular ischemia and neovascularization. At no time did he present with uveitis or vasculitis, and his serology tests were all negative. He was soon after diagnosed with sarcoidosis by hilar lymph node lung biopsy. Our patient demonstrates an atypical presentation of ocular sarcoidosis, manifesting solely as neovascularization and retinal vascular ischemia. Ophthalmologists should consider proliferative sarcoid retinopathy in patients with neovascularization.

## Introduction

Ocular involvement in sarcoidosis is common; a recent 2010 consensus study (International Workshop on Ocular Sarcoidosis or IWOS) estimated that 30-60% of sarcoid patients eventually develop ophthalmic disease [1]. Presentation of sarcoid eye disease can vary, with anterior uveitis being the most common manifestation [2]. Furthermore, the 2010 IWOS study listed the seven signs of intraocular inflammation most suggestive of ocular sarcoidosis: Mutton-fat/granulomatous keratic precipitates (KPs), trabecular meshwork nodules/tent-shaped peripheral anterior synechiae (PAS), snowballs/string-of-pearls vitreous opacities, multiple chorioretinal peripheral lesions (active/atrophic), nodular/segmented periphlebitis (± candle-wax drippings) /retinal macroaneurysms, optic disc nodules/granulomas/solitary choroidal nodule, and bilaterality [1]. 

In this report, we present a case of ocular sarcoidosis in a 59-year-old Caucasian male who presented solely as retinal neovascularization and retinal vascular ischemia.

## Case presentation

A 59-year-old white male presented with blurry vision of several months duration. His medical history was significant for hypothyroidism and a remote history of thrombophlebitis of his lower extremities and degenerative joint disease. He had a past history of smoking and no history of diabetes mellitus or hypertension. On examination, visual acuity was 20/80 in the right eye and 20/60 in the left eye. The pupillary examination was normal with no afferent pupillary defect, and the anterior segment exam was significant only for moderate nuclear sclerosis in both eyes. Intraocular pressures were within normal limits in both eyes.

**Figure 1:**
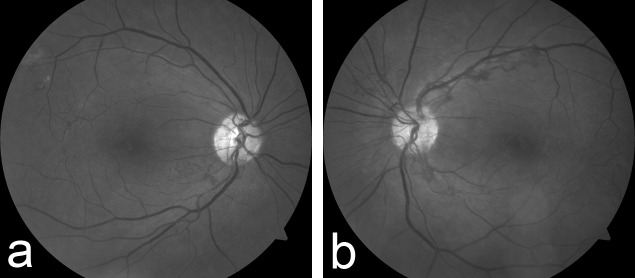
Red-Free Fundus Photography of Both Eyes on Initial Presentation Venous-venous and arteriovenous collaterals are noted in the temporal macula in the right eye (Figure 1a). Both eyes demonstrate bilateral posterior segment neovascularization, though worse in the left eye (Figure 1b).

In the right eye, venous-venous and arteriovenous collaterals were noted in the temporal macula (Figure 1a). Funduscopic examination revealed bilateral posterior segment neovascularization, worse in the left eye (Figure 1b). Mild foveal pigmentary changes were noted in both eyes. The retinal periphery was otherwise normal, and no other lesions were noted. 

**Figure 2:**
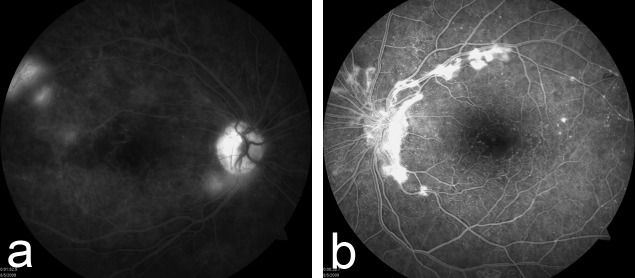
Fluorescein Angiography of Both Eyes on Initial Presentation Fluorescein angiography on presentation confirmed the presence of neovascularization bilaterally with enlarged foveal capillary avascular zones and telengiectatic vessels in both eyes (Figure 2a and Figure 2b). Figure 2a (right eye) was taken in the late phase, and Figure 2b (left eye) was taken in the early venous phase.

Fluorescein angiography confirmed the presence of neovascularization bilaterally with enlarged foveal capillary avascular zones, extending into the temporal macula in the right eye (Figure 2a). Loss of the retinal capillaries and presence of telangiectatic vessels were noted in both fundi (Figure 2a & Figure 2b). Vascular inflammatory findings were absent in both eyes.

A laboratory investigation was obtained which included a serum fluorescent treponemal antibody absorption (FTA-ABS), rapid plasma reagin (RPR), antinuclear antibody (ANA), lysozyme level, angiotensin converting enzyme (ACE) level, and coagulopathy work up; all of which were within normal limits. The patient tested negative for hepatitis C and human immunodeficiency virus (HIV). A transesophageal echocardiogram was negative for thrombi and was significant only for moderate pulmonary hypertension, moderate aortic regurgitation, and a small pericardial effusion.  Carotid ultrasonography at this time demonstrated no significant stenosis or thrombi.

At six-months post-presentation, the patient underwent panretinal photocoagulation in both eyes for new onset bilateral vitreous hemorrhage. At 16-months post-presentation, he required cataract surgery in his left eye; at 19-months, he underwent a pars plana vitrectomy and additional endolaser panretinal photocoagulation for a non-clearing vitreous hemorrhage in his left eye. At no time did the patient present with signs of uveitis or vasculitis.

**Figure 3:**
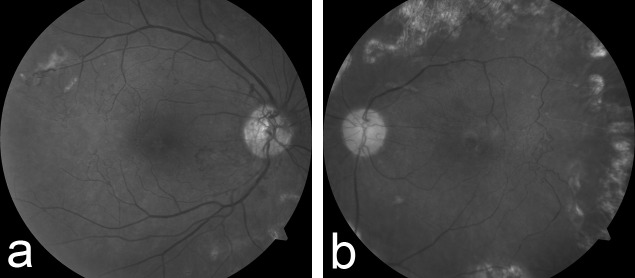
Red-Free Fundus Photography of Both Eyes 25 Months after Presentation Red-free images 25 months after presentation demonstrate worsening posterior segment neovascularization despite panretinal photocoagulation. Mature arteriovenous collaterals are noted traversing large areas of capillary dropout and vascular pruning in the temporal macular in the right eye (Figure 3a). The left eye shows regressed neovascularization, marked attenuation and sclerosis of the retinal arterial vasculature, and venous-venous collaterals (Figure 3b).

At 25-months final follow-up, his vision tested at 20/60 in the left eye and 20/100 in the right eye. At this time, funduscopic examination of the right eye demonstrated worsening posterior segment neovascularization. The arteriovenous collaterals and temporal macular vascular pruning had become more prominent. Examination of the left eye demonstrated regression of neovascularization, marked attenuation and sclerosis of the retinal arterial vasculature, and venous-venous collaterals (Figure 3a & Figure 3b).

**Figure 4:**
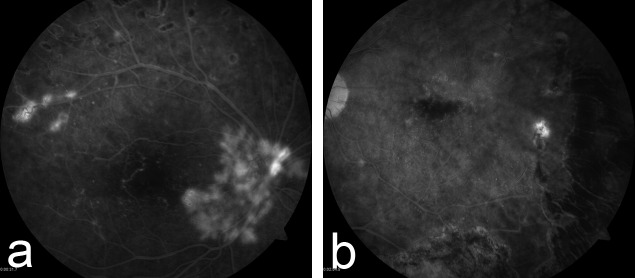
Fluorescein Angiography of Both Eyes 25 Months after Presentation Fluorescein angiography 25 months after presentation confirmed the loss of foveal capillary architecture, greater in the right eye (Figure 4a). Persistent neovascularization is also present in the right eye (Figure 4a). Late capillary leakage from telengiectatic vessels is present in the left eye (Figure 4b).

Fluorescein angiography confirmed continued loss of foveal capillary architecture, greater in the right eye. Mild residual leakage from neovascularization in the right eye and late capillary leakage in the left eye were also noted (Figure 4a & Figure 4b). Spectral domain optical coherence tomography demonstrated only mild inner limiting membrane (ILM) changes in the left eye without secondary macular thickening. The retinal contour in both eyes was within normal limits.

At this time, 25-months post-presentation, a computed tomography (CT) angiogram of the thoracic aorta, great vessels, brain and neck vessels demonstrated a 70% stenosis of the right proximal external carotid artery, but otherwise, no significant stenosis was noted involving the carotid and vertebral systems. However, a 5 mm non-calcified, right middle lobe nodule along with mediastinal adenopathy was noted. Four months after his last visit to the eye clinic, that is 29 months after presentation, the patient was admitted to the hospital for dyspnea and congestive heart failure. During this admission, a repeat CT scan demonstrated worsening hilar adenopathy, and the patient underwent a hilar lymph node biopsy. Histopathologic examination confirmed the diagnosis of sarcoidosis.

## Discussion

Isolated posterior segment involvement is noted in only 5% of patients with sarcoid disease [3]. The most common posterior findings are chorioretinitis and retinal periphlebitis with retinal hemorrhage. Roth spots, venous occlusions, and retinal neovascularization are atypical. In the largest series of sarcoid proliferative retinopathy patients, direct microvascular ischemia, rather than inflammation, has been proposed as the etiology of proliferative disease [4]. Our experience supports this theory in that no signs of retinal phlebitis or ocular inflammation were noted at any time during his clinical course, with the patient’s only retinal manifestations being progressive retinal vascular ischemia. Notably, ACE and lysozyme levels were negative. Both enzymes have limitations as neither predicts nor rules out sarcoidosis with absolute sensitivity or specificity [5-6].

## Conclusions

Ophthalmologists should be aware of the rare presentation of ocular sarcoidosis limited to retinal vascular ischemia and neovascularization.
